# Evaluation of the efficacy and safety of Melatonin in moderately ill patients with COVID-19: A structured summary of a study protocol for a randomized controlled trial

**DOI:** 10.1186/s13063-020-04737-w

**Published:** 2020-10-26

**Authors:** Ava Ziaei, Parivash Davoodian, Habib Dadvand, Omid Safa, Soheil Hassanipour, Mahmoud Omidi, Moein Masjedi, Fahime Mahmoudikia, Bahareh Rafiee, Mohammad Fathalipour

**Affiliations:** 1 Infectious and Tropical Diseases Research Center, Hormozgan Health Institute, Hormozgan University of Medical Sciences, Bandar Abbas, Iran; 2 Department of Clinical Pharmacy, Faculty of Pharmacy, Hormozgan University of Medical Sciences, Bandar Abbas, Iran; 3 Gastrointestinal and Liver Diseases Research Center, Guilan University of Medical Sciences, Rasht, Iran; 4 Department of Pharmacology and Toxicology, Faculty of Pharmacy, Hormozgan University of Medical Sciences, Bandar Abbas, Iran; 5 Department of Pharmaceutics, School of Pharmacy, Shiraz University of Medical Sciences, Shiraz, Iran; 6 Endocrinology and Metabolic Research Center, Hormozgan University of Medical Sciences, Bandar Abbas, Iran

**Keywords:** COVID-19, Randomised controlled trial, Protocol, Melatonin, Inflammatory responses, Clinical symptoms

## Abstract

**Objectives:**

We will evaluate the efficacy and safety of Melatonin, compared to the standard therapeutic regimen on clinical symptoms and serum inflammatory parameters in patients with confirmed COVID-19, who are moderately ill.

**Trial design:**

This is a single-center, randomized, double-blind, placebo-controlled clinical trial with a parallel-group design conducted at Shahid Mohammadi Hospital, Bandar Abbas, Iran.

**Participants:**

All patients admitted to Severe Acute Respiratory Syndrome Departments of Shahid Mohammadi Hospital, Bandar Abbas, Iran will be screened for the following criteria.

*Inclusion criteria*:

1. Age ≥20 years

2. Confirmed SARS-CoV-2 diagnosis (positive polymerase chain reaction).

3. Moderate COVID-19 pneumonia (via computed tomography and or X-ray imaging), requiring hospitalization.

4. Hospitalized ≤48 hours.

5. Signing informed consent and willingness of the participant to accept randomization to any assigned treatment arm.

*Exclusion criteria*:

1. Underlying diseases, including chronic hypertension, diabetes mellitus, seizure, depression, chronic hepatitis, cirrhosis, and cholestatic liver diseases.

2. Severe and critical COVID-19 pneumonia.

3. Use of warfarin, corticosteroids, hormonal drugs, alcohol, other antiviral and investigational medicines, and illegal drugs (during the last 30 days).

4. History of known allergy to Melatonin.

5. Pregnancy and breastfeeding.

**Intervention and comparator:**

*Intervention group*: The standard treatment regimen for COVID-19, according to the Iranian Ministry of Health and Medical Education's protocol, along with Melatonin capsules at a dose of 50 mg daily for a period of seven days.

*Control group*: The standard therapeutic regimen for COVID-19 along with Melatonin-like placebo capsules at a dose of one capsule daily for a period of seven days.

Both Melatonin and placebo capsules were prepared at the Faculty of Pharmacy and Pharmaceutical Sciences, Hormozgan University of Medical Sciences, Bandar Abbas, Iran.

**Main outcomes:**

The primary outcomes are the recovery rate of clinical symptoms and oxygen saturation as well as improvement of serum inflammatory parameters, including C-reactive protein, tumor necrosis factor-alpha (TNF-ɑ), interleukin-1β (IL-1β), and IL-6 within seven days of randomization.

The secondary outcomes are the time to improve clinical and paraclinical features along with the incidence of serious adverse drug reactions within seven days of randomization.

**Randomization:**

Included patients will be allocated to one of the study arms using block randomization in a 1:1 ratio (each block consists of 10 patients). This randomization method ensures a balanced allocation between the arms during the study. A web-based system will generate random numbers for the allocation sequence and concealment of participants. Each number relates to one of the study arms.

**Blinding (masking):**

All study participants, clinicians, nurses, research coordinators, and those analyzing the data are blinded to the group assignment.

**Numbers to be randomized (sample size):**

A total of 60 patients randomized into two groups (30 in each group).

**Trial Status:**

The trial protocol is Version 1.0, August 14, 2020. Recruitment began August 22, 2020, and is anticipated to be completed by November 30, 2020.

**Trial registration:**

The trial protocol has been registered in the Iranian Registry of Clinical Trials (IRCT). The registration number is “IRCT20200506047323N5”. The registration date was 14 August 2020.

**Full protocol:**

The full protocol is attached as an additional file, accessible from the Trials website (Additional file 1). In the interest in expediting the dissemination of this material, the familiar formatting has been eliminated; this Letter serves as a summary of the key elements of the full protocol.

## Supplementary information

**Additional file 1.** Full Study Protocol.

## Data Availability

The corresponding author has access to the final trial information, and the data will be available on reasonable request (Contact: M.fathalipour@hums.ac.ir).

