# Supplementary material for: Evaluation of the efficacy and safety of Melatonin in moderately ill patients with COVID-19: A structured summary of a study protocol for a randomized controlled trial
Source: Trials. 2020 Oct 26;21:882. doi: 10.1186/s13063-020-04737-w (PMC7586647; doi:10.1186/s13063-020-04737-w)
Supplement: Supplementary file 1 — Additional file 1. Full Study Protocol. [file 13063_2020_4737_MOESM1_ESM.pdf]

Protocol

This trial protocol has been provided by the authors to give readers additional information about their work.

Evaluation of the efficacy and safety of Melatonin in patients with COVID-19: A structured summary of a study protocol for a randomized controlled trial

Ava Ziaei [1], Parivash Davoodian [1], Habib Dadvand [1], Omid Safa [2], Soheil Hassanipour [3], Mahmoud Omidi [4], Fahime Mahmoudikia [1], Bahareh Rafiee [4], Mohammad Fathalipour [4,5]

[1] *Infectious and Tropical Diseases Research Center, Hormozgan Health Institute, Hormozgan University of Medical Sciences, Bandar Abbas, Iran.*

[2] *Department of Clinical Pharmacy, Faculty of Pharmacy, Hormozgan University of Medical Sciences, Bandar Abbas, Iran.*

[3] *Gastrointestinal and Liver Diseases Research Center, Guilan University of Medical Sciences, Rasht, Iran.*

[4] *Department of Pharmacology and Toxicology, Faculty of Pharmacy, Hormozgan University of Medical Sciences, Bandar Abbas, Iran.*

[5] *Endocrinology and Metabolic Research Center, Hormozgan University of Medical Sciences, Bandar Abbas, Iran.*

Corresponding author: Mohammad Fathalipour
Department of Pharmacology and Toxicology, Faculty of Pharmacy, Hormozgan University of Medical Sciences, Bandar Abbas, Iran.
Telephone: +98-9133962826
Fax: +98-7132307591
Email addresses: m.fathalipour@Hums.ac.ir

Abstract

No effective treatment has yet been proven for the coronavirus disease 2019 (COVID-19). Melatonin is one of strong anti-inflammatory agents that had been effectively used in the management of a number of inflammatory diseases. The present study aims to evaluate efficacy and safety of Melatonin in patients with COVID-19.

A perspective randomized placebo-control clinical trial will be conducted on 60 hospitalized adult patients with covid-19 positive polymerase chain reaction test. Patients will be randomly assigned in a 1:1 ratio to receive either Melatonin (50 mg once a day) along with standard care (lopinavir–ritonavir 200/50 mg twice a day and hydroxychloroquine 200 mg twice a day) for seven days, or placebo and standard care alone.

The primary outcomes are the recovery rate of clinical symptoms and oxygen saturation as well as improvement of serum inflammatory parameters, including C-reactive protein, tumor necrosis factor-alpha (TNF-α), interleukin-1β (IL-1β), and IL-6. The secondary outcomes are the time to improve clinical and paraclinical features and the incidence of serious adverse drug reactions.

بررسی اثربخشی و ایمنی ملاتونین در بیماران مبتلا به COVID-19: یک کارآزمایی بالینی تصادفی

چکیده

هنوز هیچ درمان موثری برای بیماری کورنا ویروس ۲۰۱۹ (COVID-19) تائید نشده است. ملاتونین یکی از عوامل ضد التهابی قوی است که به طور موثری در مدیریت تعدادی از بیماری های التهابی استفاده شده است. مطالعه حاضر با هدف ارزیابی کارایی و ایمنی ملاتونین در بیماران مبتلا به COVID-19 انجام خواهد شد.

یک کارآزمایی بالینی تصادفی کنترل شده با پلاسبو ، روی ۶۰ بیمار بزرگسال بستری با تست مثبت واکنش زنجیره ای پلیمراز برای کووید ۱۹ انجام خواهد شد. بیماران به صورت تصادفی به نسبت ۱: ۱ در گروه های ملاتونین (۵۰ میلی گرم یک بار در روز) همراه با درمان استاندارد (لوپیناویر-ریتوناویر ۵۰/۲۰۰ میلی گرم دو بار در روز و هیدروکسی کلروکین ۲۰۰ میلی گرم دو بار در روز) و یا گروه دارو نما و درمان استاندارد تقسیم می شوند.

پیامد های اصلی شامل بهبودی علائم بالینی و اشباع اکسیژن و همچنین بهبود پارامترهای التهابی سرم ، از جمله پروتئین واکنش پذیر C ، فاکتور نکروز تومور آلفا (TNF-α) ، اینترلوکین-۱(IL-1β) β و IL -6 خواهند بود. نتایج ثانویه نیز شامل زمان بهبود علائم بالینی و پاراکلینیکی و بروز واکنش های جانبی جدی دارو است.

مقدمه

از ۸ دسامبر سال ۲۰۱۹ ، موارد متعددی از پنومونی ناشی از بیماری ناشناخته در ووهان ، استان هوبی چین گزارش شده است. بیشتر بیماران در بازار عمده فروشی غذاهای دریایی هوآنان کار می کردند یا زندگی می کردند ، جایی که حیوانات زنده نیز فروخته می شدند [۱]. مشخص شد که بیماران یک کورونا ویروس جدیدی دارند که از نظر ژنتیکی مربوط به خفاش کروناویروس است که به آن SARS-CoV-2 گفته شد [۲]. در زمان نوشتن این پروپوزال طبق آمار WHO بیش از هفت میلیون مورد از افراد آلوده و ۲۱۶ کشور و بیش از ۴۰۰۰۰۰ مورد مرگ ناشی از این بیماری گزارش شده است.

به طور خلاصه، دو ویروس والد SARS-CoV-2 اکنون شناسایی شده اند. اولین مورد ، bat coronavirus RaTG13است که در Rhinolophus affinis از استان یونان یافت می شود و ۹۶,۲٪ توالی ژنتیکی مشابهی با SARS-CoV-2 دارد [۳]. مورد دوم ، گروهی از بتاکورونا ویروسها است که در گونه های در معرض خطر پستانداران کوچک مشهور به پنگولین ها یافت می شود. این ویروس ها با کورونا ویروس جدید ۹۰٪ توالی نوکلئوتیدی مشابه و۹۷,۴٪ توالی آمینو اسید مشابه در زنجیره متصل شونده به گیرنده ی ACE دارند [۳].

بیشتر بیماران مبتلا به COVID-19 علائم خفیف تا متوسط را نشان می دهند ، اما تقریباً ۱۵٪ تا پنومونی شدید پیش می روند و حدود ۵٪ در نهایت دچار سندرم حاد تنفسی (ARDS) ، شوک سپتیک و / یا نارسایی اندام های متعدد می شوند. پایه اصلی درمان بالینی شامل مدیریت علائم و درمان با اکسیژن و تهویه مکانیکی برای بیماران دارای نارسایی تنفسی است. اگرچه چندین داروی ضد ویروسی ، از جمله نوکلئوتید آنالوگ رمسیدور ، به طور فعال آزمایش می شوند ، اما هیچکدام به طور خاص برای COVID-19 تأیید نشده است. علاوه بر توسعه واکسن و رویکردهایی که به طور مستقیم ویروس را هدف قرار داده و یا ورود ویروسی را مسدود می کند ، درمان هایی که ایمونوپاتولوژی عفونت را مورد توجه قرار می دهند ، به کانون اصلی توجه تبدیل شده اند [۴]. در بیماران مبتلا به COVID-19 شدید ، اما نه در بیماران مبتلا به بیماری خفیف ، لنفوپنی یکی از ویژگیهای متداول است

، همراه با کاهش چشمگیر سلولهای CD4 + T ، سلولهای CD8 + T ، سلولهای B و سلولهای کشنده طبیعی (NK) [5] و همچنین کاهش درصد مونوسیت ها ، ائوزینوفیل ها و بازوفیل ها [۶] بیشتر بیماران مبتلا به COVID-19شدید ، سطح سرمی قابل توجهی از سایتوکاین های التهابی از جمله IL-6 و IL-1β وIL-2 ، IL-8 ، IL-17 ، G-CSF، GM-CSF، (CCL3) MIP1α MCP1, IP10,را نشان می دهند که این پدیده به عنوان cytokine stormشناخته می شود [۷] مقادیر زیاد سیتوکین های التهابی ممکن است منجر به شوک و آسیب بافتی در قلب ، کبد و کلیه و همچنین نارسایی تنفسی یا نارسایی اندام های متعدد شود. آنها همچنین با واسطه آسیب ریوی گسترده ، منجر به نفوذ گسترده نوتروفیل ها و ماکروفاژها ، alveolar infiltrationبا تشکیل غشاهای هیالینی و ضخیم شدن دیواره آلوئول می شوند. آتروفی طحال و نکروز غدد لنفاوی نیز مشاهده شده است ، که نشانگر آسیب به واسطه ی سیستم ایمنی در بیماران متوفی است [۵].

در ICU ، آرام بخش عمیق با افزایش مرگ و میر طولانی مدت همراه است، و استفاده از ملاتونین باعث کاهش استفاده از آرامبخشی و دفعات درد و اضطراب می شود [۸]. همچنین ، یک متاآنالیز اخیر نشان داد که ملاتونین باعث بهبود کیفیت خواب در بیماران در بخش مراقبت های ویژه می شود [۹].

ملاتونین (N-استیل-۵-متیوکسی تریپتامین) یک مولکول فعال زیستی با مجموعه ای از خواص تقویت کننده سلامتی است. ملاتونین با موفقیت در درمان اختلالات خواب ، دلیریوم ، آترواسکلروز ، بیماری تنفسی و عفونت های ویروسی مورد استفاده قرار گرفته است [۱۰]. تحقیقات قبلی اثرات مثبت ملاتونین را در کاهش استرس حاد تنفسی ناشی از ویروس ، باکتریها ، پرتودرمانی و غیره را مستند کرده است [۱۱-۱۳]. در شرایط مزمن مانند دیابت و مولتیپل اسکلروز مصرف ملاتونین به ترتیب به مدت ۸ هفته و ۶ ماه به طور قابل توجهی سطح سرمی TNF-α، IL-6، IL-1β و CRP را کاهش داده است [۱۴, ۱۵]. در شرایط حاد مانند استرس جراحی ، خونرسانی مجدد مغز و خونرسانی مجدد شریان کرونر استفاده روزانه از داروی خوراکی ملاتونین ۱۰ mg / d، 6mg / d و mg / d۵ به ترتیب به طور قابل توجهی سطح سرمی سیتوکین های التهابی را کاهش می دهد [۱۶-۱۸]. همانطور که قبلاً مورد بررسی قرار گرفت ، استفاده کوتاه مدت از ملاتونین بی خطر است ، حتی در موارد مصرف

زیاد ، و عوارض جانبی گزارش شده محدود به سرگیجه ، سردرد ، حالت تهوع و خواب آلودگی هستند. به طور کلی ایمنی ملاتونین در انسان بسیار بالا است [۱۹]. در آزمایشات بالینی ، دوزهای ۳ میلی گرم ، ۶ میلی گرم و ۱۰ میلی گرم خوراکی ملاتونین خوراکی توسط بیماران در بخش مراقبت های ویژه ، در مقایسه با دارونما ، ایمنی رضایت بخش نشان داد [۹]. همچنین ، حتی وقتی ملاتونین به مدت دو ماه به میزان ۱ گرم در روز به انسان تجویز شد ، هیچ گزارش نامطلوبی از درمان گزارش نشده است [۲۰]. سرانجام ، هیچ عارضه جانبی پس از استفاده از ملاتونین در مطالعات حیوانی ALI / ARDS ثبت نشده است [۹]. به همین ترتیب و با توجه به عدم وجود یک کارآزمایی بالینی در مورد تأثیر ملاتونین بر بیماران covid-19، تصمیم به اجرای این کارآزمایی در بیمارستان شهید محمدی بندرعباس، هرمزگان، ایران گرفتیم.

بررسی متون

تظاهرات معمول COVID-19 شامل تب ، سرفه خشک ، خستگی ، بی اشتهایی ، بو و اختلال در چشایی ، میالژی و تنگی نفس است. تظاهرات کمتر شایع شامل آبریزش بینی ، سردرد ، حالت تهوع ، اسهال ، گلودرد و تظاهرات پوستی می باشد. به طور فزاینده ، تظاهرات پوستی ، بثورات و ضایعات اریتماتو در اندامها گزارش شده است ، خصوصاً در موارد خفیف [۲۱].

اولین گزارش های مربوط به عفونت ها در چین حاکی از آن است که ۸۱٪ موارد خفیف و ۱۴٪ شدید هستند و به مراقبت های حمایتی نیاز دارند و ۵٪ منجر به بیماری بحرانی می شوند [۲۲].

در برخی موارد ، پیشرفت شدید بیماری منجر به ALI / ARDS ، نارسایی تنفسی ، نارسایی قلبی ، سپسیس و ایست قلبی ناگهانی در طی چند روز می شود [۱, ۵].

شروع ARDS به طور متوسط ۱۰,۵ روز پس از شروع علائم در یک مطالعه اتفاق افتاد [۵].

بر اساس ویژگی های بالینی ، پاتولوژی ، پاتوژنز اختلال تنفسی حاد ناشی از آلودگی با کورونا ویروس ها ی بسیار مشابه یا سایر عوامل بیماری زا ، شواهد نشان می دهد که التهاب بیش از حد ، اکسیداسیون و پاسخ ایمنی اغراق آمیز به احتمال زیاد در COVID-19 نقش دارد. این منجر به طوفان سیتوکین و پیشرفت متعاقب آن به آسیب حاد ریه (ALI/ARDS) و اغلب مرگ می شود [8].

در مراحل اولیه عفونت کورونا ویروس ها ، سلول های دندریتیک و سلول های اپیتلیال فعال شده و خوشه ای از سیتوکین های التهابی و کموکاین ها از جمله IL-1β، IL-2، IL-6، IL-8 را نشان می دهند ، هر دو IFN-α / β ، TNF، موتیف کموکاین ۳ (CCL3)، CCL5، CCL2 و IP-10 و غیره. اینها تحت کنترل سیستم ایمنی هستند. بنابراین ، تولید بیش از حد این سیتوکین ها و کموکاین ها به پیشرفت در بیماری کمک می کند [۲۳-۲۵].

فیزیولوژی عفونت COVID-19 و علت اصلی مرگ در بیماران آلوده به این ویروس شامل یک التهاب تشدید شده (همراه با نفوذ سلول های ایمنی ، نکروز ، و هایپرپلازی بافت مبتلا) به خصوص در سطح ریه است. این منجر به اختلال در تبادل اکسیژن ریوی و نومونی شدید می شود [۲۶].

در خون بیماران مبتلا به COVID-19 ، افزایش قابل توجهی در اینترلوکین IL-1β، اینترفرون (IFN-γ) γ ، پروتئین القایی اینترفرون (IP-10)وmcp-1 ، IL-4 و IL-10 مشاهده شد [۵].

گزارش های اخیر حاکی از آن است که در برخی از بیماران COVID-19 ، اگرچه برای آزمایش اسید نوکلئیک ویروسی منفی است ، اما هنوز هم گاهی اوقات سطح بالایی از التهاب وجود دارد [۸].

ملاتونین ضد ویروس نیست اما به دلیل ویژگی های ضد التهاب ، ضد اکسیداسیون و تقویت کننده سیستم ایمنی بدن ، دارای خواص ضد ویروس به صورت غیرمستقیم است [۸].

در موشهایی که سیستم عصبی مرکزی آنها به ویروس آلوده شده است (به عنوان مثال به آنسفالیت مبتلا شده اند) ، استفاده از ملاتونین باعث ایجاد viremia کمتر ، کاهش فلج و مرگ و کاهش viral load شد [۲۷].

در مدلهای قبلی ویروس سینسیتیال تنفسی ، ملاتونین باعث کاهش آسیب اکسیداتیو حاد ریه ، انتشار سیتوکین التهابی و فراخوانی سلولهای التهابی شد [۱۳].

احمد زارع و همکاران در یک تحقیق در سال ۲۰۲۰ نشان دادند که در بیماران دیابتی که از بیماری پریودنتال رنج می برند ، تجویز خوراکی روزانه ۲۵۰ میلی گرم ملاتونین به طور قابل توجهی سطح سرمی IL-1B و MDA(مالون دی آلدئید) را کاهش و سطح سوپر اکسید دیسموتاز(SOD)، کاتالاز،ظرفیت کلی آنتی اکسیدانتی (TAC) و گلوتاتیون پراکسیداز را افزایش می دهد [۲۸].

در یک مطالعه in vivo توسط کارراسکو و همکاران در سال ۲۰۱۳ ، تزریق ۲۵ میلی گرم بر کیلوگرم ملاتونین در پانکراتیت ناشی از کرووئین به طور قابل توجهی آسیب بافتی و سطح سرمی سیتوکین های التهابی IL-1B و TNF-α را کاهش داده و باعث افزایش سیتوکین ضد التهابی IL-4 شد [۲۸].

در مطالعه ی مروری که به تازگی توسط پورهنیفه و همکاران در سال ۲۰۲۰ انجام شد ، ملاتونین خواص پیشگیری کننده از عوارض دیابتی متعدد از جمله رتینوپاتی دیابتی ، نوروپاتی کاردیومیوپاتی و نفروپاتی را به دلیل فعالیت ضد التهابی ، ضد اکسید کننده و scavengering نشان می دهد [۲۹].

در بررسی ای که اخیراً توسط کوپولا و همکاران انجام شده است نشان داده شد که ریتم شبانه روزی متغیرهای فیزیولوژیکی متعددی از جمله چرخه خواب بیدار ، دمای بدن ، ضربان قلب ، فشار خون ، ترشح هورمون ها ، متابولیسم و سیستم ایمنی را تحت تأثیر قرار می دهد. ریتم شبانه روزی در ICU با تغییر در الگوی طبیعی ۲۴ ساعت ترشح ملاتونین و کورتیزول و به دلیل اختلال خواب به شدت مختل می شود. کمبود خواب با اختلال در سیستم ایمنی و قلبی عروقی ، متابولیسم گلوکز ، استقامت عضلات ، حافظه و شناخت همراه است [۳۰].

Akbulut و همکاران در مطالعه ای بر روی موش ها نشان دادند که استرس اکسیداتیو در موش هایی که تحت CLP (cecal ligation and puncture) قرار میگیرند و به مدت ۱۰ روز ه قرار گرفتن در معرض نور ۲۴ ساعته از ملاتونین محروم میشوند، افزایش میابد. بر این اساس ، محرومیت از نور به دلیل افزایش سطح ملاتونین در بیماران بخش مراقبت های ویژه در درمان سپسیس مؤثر است [۳۱].

در مطالعه‌ای که توسط Meltem Gunur Can در سال ۲۰۱۸ بر روی بیماران کاندید برای CABG(پیوند بای پس عروق کرونر) انجام شد ، premedication ملاتونین بر خلاف بیمارانی که Alperazolam دریافت کرده بودند اثرات مثبتی بر پارامترهای آنتی اکسیدانی داشت. اگرچه اختلاف معنی داری در رابطه با سطح پارامتر استرس اکسیداتیو وجود نداشت ، اما مدت زمان خارج کردن لوله ، طول مدت بستری در بیمارستان ، مدت

زمان cross-clamp ، بای پس قلبی ریوی و عملکرد و نتایج Mini-Mental State Examination بین دو گروه وجود داشت [۳۲].

گوپتا و همکارانش در یک مطالعه در سال ۲۰۱۶ نشان دادند که داروی ملاتونین ۲ ساعت قبل از لارنگوسکوپی و لوله‌گذاری به طور قابل توجهی پاسخهای همودینامیکی از جمله فشار خون سیستولیک و دیاستولیک را بر خلاف گروه کنترل کاهش داده است [33].

در مطالعه‌ای که در سال ۲۰۱۱ توسط نیک‌خولق و همکاران انجام شد ، ۵۰ میلی گرم بر کیلو گرم وزن بدن ملاتونین در شیر حل شد و پس از لوله گذاری برای بیهوشی عمومی از طریق لوله معده تزریق شد. نتایج نشان داد که درمان ملاتونین منجر به کاهش سطح ترانس آمینازها بعد از عمل در طول دوره مطالعه شده است ، هیچ عارضه جانبی جدی در بیماران پس از درمان ملاتونین مشاهده نشده است و گرایش به ماندن کوتاهتر از ICU و ماندن در بیمارستان بعد از درمان ملاتونین وجود دارد. بنابراین ، یک دوز enteral از ملاتونین قبل از عمل به طور موثری جذب می شود و در بیمارانی که تحت عمل جراحی عمده کبدی قرار دارند ایمن است و به خوبی تحمل می شود [۳۴].

نوروزی و همکاران نشان دادند که مصرف ۳ میلی گرم ملاتونین قبل از عمل شکمی در مقایسه با گروه کنترل اضطراب ، sedation و دوز پروپوفول را کاهش می دهد [۳۵].

در مطالعه ای که توسط Andersen و همکاران انجام شد ، یک دوز بزرگ از ۱۰۰ میلی گرم ملاتونین برای تعیین عوارض آن به صورت داخل وریدی تزریق شد. نتایج نشان داد که ملاتونین داخل وریدی با دوز بالا باعث آرام بخشی نمی شود. هیچ عوارض جانبی در مطالعه گزارش نشده است [۳۶].

در یک کارآزمایی بالینی دیگر توسط گالی و همکاران نشان داده شد که ملاتونین در دوز ۱۰۰ میلی گرم هیچ گونه عارضه جانبی ندارد اما در شرایط ex vivo اثرات مفیدی بر عملکرد میتوکندری ناشی از سپسیس ، استرس اکسیداتیو و پاسخ سیتوکین در غلظت های مشابه در شرایط داخل بدن داشته است [۳۷].

Alamili و همکارانش در مطالعه مدل انسانی برای اندوتوکسمی نشان دادند که تجویز ۸ ساعته ۱۰۰ میلی گرم ملاتونین باعث کاهش سطح سرمی IL-1β و YKL-40 شد [۳۸].

در یک مطالعه ایمنی بالینی توسط Weishaupt و همکاران ، تجویز ملاتونین رکتال با دوز بالا (۳۰۰ میلی گرم در روز) به خوبی در طی یک دوره مشاهده تا ۲ سال تحمل شد. مهمتر اینکه ، پروتئین کربونیل های سرم در گردش ، که یک مارکر جانشین برای استرس اکسیداتیو فراهم می کنند ، در بیماران مبتلا به ALS زیاد شده بودند ، اما در مداخله با ملاتونین نرمال شدند [۳۹].

اهداف کلی طرح:

- بررسی اثربخشی و ایمنی ملاتونین در بیماران مبتلا به COVID-19

اهداف ویژه‌ی طرح:

- بررسی اثرات ملاتونین برعلائم بالینی در بیماران مبتلا بهCOVID-19
- بررسی اثرات ملاتونین بر یافته های آزمایشگاهی در بیماران مبتلا بهCOVID-19
- بررسی اثرات ملاتونین بر پارامتر های التهابی سرم در بیماران مبتلا بهCOVID-19
- بررسی اثرات ملاتونین بر مدت زمان بستری در بیماران مبتلا بهCOVID-19
- بررسی سیمت و عوارض جانبی ملاتونین در بیماران مبتلا به COVID-19

اهداف کاربردی طرح:

در صورت مشاهده اثرات بالینی می توان از فرم نبولایزرملاتونین، می توان از این دارو در کاهش شدت بیماری COVID-19استفاده نمود. همچنین در صورت تایید موثر بودن این دارو می توان از آن در درمان سرپایی بیماران بهره برد.

- انتظار می رود که این مطالعه به تدوین پروتکل درمانی در بیماری COVID-19 کمک کند.
- انتظار می رود که این مطالعه منجر به کاهش مشکلات درمانی دربیماری COVID-19 شود.
- انتظار می رود که نتایج این تحقیق منجر به راه کارهایی جهت کنترل بیماری COVID-19 در کشور شود.
- انتظار می رود که نتایج این تحقیق از طریق کاهش مشکلات بالینی منجر به کاهش هزینه های درمانی در بیماران COVID-19 شود.

فرضیات یا سوالات پژوهش (باتوجه به اهداف طرح):

- تجویز ملاتونین باعث بهبود علائم بالینی در بیماران مبتلا به COVID-19 می شود.

- تجویز ملاتونین باعث بهبود یافته های آزمایشگاهی در بیماران مبتلا به COVID-19 می شود.
- تجویز ملاتونین باعث بهبود پارامتر های التهابی سرم در بیماران مبتلا به COVID-19 می شود.
- تجویز ملاتونین باعث کاهش مدت زمان بستری در بیماران مبتلا به COVID-19 می شود.
- تجویز ملاتونین در بیماران مبتلا به COVID-19 به خوبی تحمل می شود.

روش اجرای طرح

طراحی مطالعه و شرکت کنندگان

این مطالعه در قالب یک کارآزمایی بالینی تصادفی با برچسب باز بر روی ۶۰ بیمار مبتلا به بیماری COVID-19 بستری شده در بخش سندرم حاد تنفسی بیمارستان شهید محمدی شهرستان بندرعباس، ایران طراحی شده است. بیماران از ۲۵ ژوئیه ۲۰۲۰ به صورت آینده نگر وارد مطالعه و پیگیری خواهند شد. با توجه به نسبت ۱:۱ بین گروه مداخله (پرتکل استاندارد کشوری به همراه کپسول های ملاتونین) و گروه کنترل (پرتکل استاندارد کشوری به همراه دارونما)، بیماران به صورت تصادفی وارد هر یک از بازو های مداخله می شوند.

در شروع مطالعه بیماران بر اساس علائم بالینی و یافته های پاراکلینیکی در گروه های خفیف، متوسط، شدید و بحرانی تقسیم بندی می شوند (جدول ۱). معیارهای ورود بیماران شامل؛ (۱) سن ۲۰ سال یا بالاتر، (۲) رضایت آگاهانه و داوطلبانه، (۳) علائم بالینی اولیه و (۴) تشخیص قطعی بیماری COVID-19 از طریق تست PCR و یا Chest CT-scan به همراه شدت متوسط خواهند بود. تمامی بیماران با سابقه ای از (۱) هپاتیت مزمن، سیروز کبدی، بیماری های کلستاتیک کبد، التهاب کیسه صفرا، (۲) مصرف دارو های ضد انعقاد همچون وارفارین، دارو های هرمونی، الکل و داروهای غیر مجاز در طی ۳۰ روز گذشته و (۳) بیماران با سابقه ی فشار خون بالا و دیابت، (۴) افراد با سابقه ی صرع و افسردگی و (۵) زنان در دوران بارداری و شیردهی از مطالعه خارج می شوند.

در ابتدای کارآزمایی، مشخصات عمومی، مشخصات دموگرافیک و سوابق پزشکی بیماران با استفاده از پرسشنامه جمع آوری می شود. پس از ارائه توضیحات کافی و کسب رضایت آگاهانه کتبی از جانب بیمار یا بستگان درجه یک (در بیماران با سطح هوشیاری پایین یا زوال عقل)، بیماران با استفاده از روش تصادفی سازی در گروه های مورد مطالعه (گروه مداخله و گروه کنترل) تقسیم می شوند.

گروه های مداخله

گروه کنترل شامل بیمارانی هستند که درمان استاندارد بر اساس پروتکل تعیین شده از سوی وزارت بهداشت برای بیماری COVID-19 (دارو هیدروکسی کلروکین به میزان ۲۰۰ میلی گرم دو بار در روز به مدت ۷ روز) به همراه دارونما را دریافت می کنند. گروه مداخله نیز شامل بیمارانی هستند که علاوه بر درمان استاندارد فوق الذکر، ملاتونین (۵۰میلی گرم یک بار در روز به مدت ۷ روز) دریافت خواهند کرد. با توجه به اینکه فرمولاسیون های ملاتونین با دوز مدنظر در بازار دارویی موجود نمی باشد، پودر ملاتونین با همکاری شرکت داروسازی وانا دارو تهیه می شود و در دانشکده ی داروسازی دانشگاه علوم پزشکی هرمزگان، به صورت کپسول های ۵۰ میلی گرمی فرموله می شوند. دارونما مورد استفاده در این مطالعه نیز به صورت کپسول با همان شکل ظاهری و با استفاده از پودر نخودچی تهیه می شود.

بررسی پیامد ها

پیامد های اولیه این مطالعه میزان بهبودی علائم بالینی در طی دوره مداخله نظر گرفته می شود. بهبود علائم بالینی به عنوان بهبود مداوم (بیشتر از ۷۲ ساعت) درجه حرارت بدن، فرکانس تنفس و میزان اشباع اکسیژن خون پس از شروع درمان می باشد که با معیارهای کمی که در ادامه آمده است تعریف می شود: دمای دهانی ≤ ۳۶/۶ درجه سانتیگراد؛ فرکانس تنفسی ≤۲۴ بار در دقیقه و اشباع اکسیژن ≥۹۸ درصد بدون تنفس مکانیکی. علاوه بر این، علائم بالینی دیگر علائم بالینی دیگر همچون سرفه، درد عضلانی، سردرد، تنگی نفس، ضعف و بی حالی، کاهش حس بویایی و چشایی، اسهال، اسپاسم شکمی، تهوع و استفراغ به صورت کیفی در فالوآپ های روزانه ثبت می شوند. اندازه گیری مکرر حداقل برای دو بار در هر فالوآپ انجام می شود.

پیامد های ثانویه شامل مدت زمان بستری بودن در بیمارستان، فاصله زمانی از شروع مطالعه (تصادفی سازی نمونه ها در هر یک از گروه های مطالعه) تا کاهش تب، تسکین سرفه و بهبود تنگی نفس و میزان نیاز اکسیژن درمانی کمکی یا تهویه مکانیکی غیر تهاجمی در طول مطالعه، نیاز به بستری شدن در بخش مراقبت های ویژه و میزان نارسایی تنفسی در طول مطالعه) اشباع اکسیژن ≤۹۰ درصد بدون تنفس مکانیکی و یا PaO2/FiO2 <300 میلی متر جیوه، نیاز به اکسیژن درمانی و یا حمایت تنفسی (ثبت می شوند.

انجام آزمایش های CBC[۴]، شمارش تفریقی سلول های سفید، میزان , IL-1B, IL-6, TNF-α، D-dimer، CRP، LDH، ESR، کراتینین و نیتروژن اوره سرم در ابتدای مطالعه و انتهای مطالعه (روز ۷ مطالعه و یا زمان ترخیص) بررسی خواهد شد. علاوه بر این تست PCR (و یا Chest CT-scan) و انتهای مطالعه (روز ۷ مطالعه و یا زمان ترخیص) بررسی خواهد شد. همچنین واکنش های نا خواسته ، فراوانی عوارض جانبی احتمالی ناشی از مداخله و فراوانی انصراف از مطالعه به علت عوارض جانبی در گروه های مورد مطالعه به صورت روزانه ثبت می شوند. شایع ترین عوارض دارویی ملاتونین شامل سردرد، سرگیجه، حالت تهوع، خواب آلودگی، فشار خون پایین و خواب آلودگی در روز می باشد. بیماران مصرف کننده داروهای القاء یا مهارکننده آنزیم های به طور مستمر مورد پایش قرار می گیرند و در صورت بروز تداخل و تغییر یا احتمال تغییر در پاسخ بیماران به دارو ها، مصرف ملاتونین قطع خواهد شد. بیماران از نظر فشار خون به طور مستمر مورد پایش قرار خواهند گرفت و در صورت افزایش فشار خون تجویز ملاتونین قطع خواهد شد.

روش محاسبه حجم نمونه

استفاده از مطالعات مشابه انجام شده در گروه های چند دارویی میزان بهبودی بالینی مورد انتظار در روز ۷ از گروه مورد مطالعه ۸۰ درصد، بهبودی بالینی گروه دریافت کننده دارونما ۴۰ درصد، $\alpha = 0.05$ ، $\beta = 0.10$، قدرت = ۰/۹۰ در نظر گرفته شد. با توجه به توزیع ۱:۱ بین گروه مورد مطالعه و گروه دریافت کننده دارونما، حجم نمونه آماری ۲۴ شرکت کننده در هر گروه است. اندازه نمونه با توجه به عواملی مانند ریختن حدود ۲۰ درصد افزایش یافته است. این کارآزمایی شامل ۶۰ نفر (۳۰ نفر در هر گروه) می باشد.

روش تصادفی سازی بلوک

در این روش تعداد افراد در هر یک از گروه های مطالعه در طول درمان با یکدیگر برابر است. با توجه به تعداد ۶۰ نفر افراد شرکت کننده (۳۰ نفر در هر گروه) و مدت زمان تقریبی ۱۰ هفته برای تکمیل ورود افراد به مطالعه، از ۱۰ بلوک ۶ تایی (در صورت وجود بیمار به تعداد کافی از ۵ بلوک ۱۲ تایی) استفاده خواهد شد. روش کار در این

نوع از تصادفی سازی شبیه به روش تصادفی سازی ساده است، تنها تعداد افراد در طول دوره درمان در دو گروه مداخله و کنترل یکسان می باشد. تنها ایراد این روش مشخص شدن آخرین گروه در هر بلوک می باشد.

آنالیز آماری

برای تحلیل آماری از نرم افزار SPSS ورژن ۱۸/۰ استفاده می شود. برای مقایسه شاخص های اصلی اثر بخشی (میزان بهبود علائم بالینی) به عنوان پیامد اولیه و پیامد های ثانویه بین گروه مورد مطالعه و گروه دریافت کننده دارونما، از آزمون t برای متغیرهای پیوسته و یا آزمون Wilcoxon (در صورت عدم استفاده از آزمون t) برای متغییر های گسسته رتبه ای استفاده می شود. توصیف آماری متغیرهای کیفی به صورت فراوانی یا درصد مشاهده خواهد بود و برای مقایسه بین گروه ها از آزمون های Chi-square یا Fisher’s exact استفاده خواهد شد. برای کلیه آزمون های آماری، $P < 0.05$ (دو طرفه) از نظر آماری معنی دار در نظر گرفته می شود.

ملاحظات اخلاقی

جهت شرکت در این مطالعه از تمامی بیماران رضایت آگاهانه اخذ خواهد شد. اطلاعات مربوطه بصورت محرمانه حفظ خواهد شد. هیچ یک از مشخصات فردی افراد شرکت کننده از جمله اسم و فامیل آنها وارد کامپیوتر نخواهد شد و به تمامی افراد کد پروژه داده شده و آنالیز بر اساس آن انجام خواهد شد. اطلاعات اولیه در فایل های قفل دار و نزد مجری اصلی تا اتمام پروژه و انتشار مقالات باقی خواهد ماند. در هر مرحله از طرح بیماران می توانند بنا به تمایل شخصی از طرح خارج شوند.

محدودیت‌های اجرایی طرح و روش رفع آن ها

عدم همکاری بیماران که امید است با توضیحات کافی برای آن ها این مشکل رفع شود و قبل از شروع به بیماران در مورد عوارض جانبی بیماری و دارو توضیح داده می شود. از آنها قبل از شروع درمان رضایت نامه کتبی دریافت می شود. همچنین این پژوهش از نظر اخلاق پزشکی مورد تایید است.

References

1. Chen, N., et al., *Epidemiological and clinical characteristics of 99 cases of 2019 novel coronavirus pneumonia in Wuhan, China: a descriptive study.* Lancet, 2020. **395**(10223): p. 507-513.
2. Khan, M.M., et al., *Emergence of novel coronavirus and progress toward treatment and vaccine.* Rev Med Virol, 2020.
3. Yuen, K.S., et al., *SARS-CoV-2 and COVID-19: The most important research questions.* Cell Biosci, 2020. **10**: p. 40.
4. Cao, X., *COVID-19: immunopathology and its implications for therapy.* Nature reviews. Immunology, 2020. **20**(5): p. 269-270.
5. Huang, C., et al., *Clinical features of patients infected with 2019 novel coronavirus in Wuhan, China.* Lancet, 2020. **395**(10223): p. 497-506.
6. Qin, C., et al., *Dysregulation of Immune Response in Patients With Coronavirus 2019 (COVID-19) in Wuhan, China.* Clinical Infectious Diseases, 2020.
7. Tan, M., et al., *Immunopathological characteristics of coronavirus disease 2019 cases in Guangzhou, China.* Immunology, 2020.
8. Zhang, R., et al., *COVID-19: Melatonin as a potential adjuvant treatment.* Life Sci, 2020. **250**: p. 117583.
9. Lewis, S.R., et al., *Melatonin for the promotion of sleep in adults in the intensive care unit.* Cochrane Database of Systematic Reviews, 2018(5).
10. Reiter, R.J., Q. Ma, and R. Sharma, *Treatment of Ebola and other infectious diseases: melatonin "goes viral".* Melatonin Research, 2020. **3**(1): p. 43-57.
11. Wu, X., et al., *Melatonin alleviates radiation-induced lung injury via regulation of miR-30e/NLRP3 Axis.* Oxidative medicine and cellular longevity, 2019. **2019**.
12. Yip, H.K., et al., *Melatonin treatment improves adipose‐derived mesenchymal stem cell therapy for acute lung ischemia–reperfusion injury.* Journal of pineal research, 2013. **54**(2): p. 207-221.
13. Huang, S.H., et al., *Inhibitory effect of melatonin on lung oxidative stress induced by respiratory syncytial virus infection in mice.* Journal of pineal research, 2010. **48**(2): p. 109-116.
14. Bazyar, H., et al., *The effects of melatonin supplementation in adjunct with non-surgical periodontal therapy on periodontal status, serum melatonin and inflammatory markers in type 2

diabetes mellitus patients with chronic periodontitis: a double-blind, placebo-controlled trial. Inflammopharmacology, 2019. **27**(1): p. 67-76.

15. Sánchez-López, A.L., et al., *Efficacy of melatonin on serum pro-inflammatory cytokines and oxidative stress markers in relapsing remitting multiple sclerosis.* Archives of medical research, 2018. **49**(6): p. 391-398.

16. Zhao, Z., et al., *The protective effect of melatonin on brain ischemia and reperfusion in rats and humans: In vivo assessment and a randomized controlled trial.* Journal of pineal research, 2018. **65**(4): p. e12521.

17. Shafiei, E., et al., *Effects of N-acetyl cysteine and melatonin on early reperfusion injury in patients undergoing coronary artery bypass grafting: A randomized, open-labeled, placebo-controlled trial.* medicine, 2018. **97**(30).

18. Kücükakin, B., et al., *Utility of melatonin to treat surgical stress after major vascular surgery–a safety study.* Journal of pineal research, 2008. **44**(4): p. 426-431.

19. Andersen, L.P.H., et al., *The safety of melatonin in humans.* Clinical drug investigation, 2016. **36**(3): p. 169-175.

20. NORDLUND, J.J. and A.B. LERNER, *The effects of oral melatonin on skin color and on the release of pituitary hormones.* The Journal of Clinical Endocrinology & Metabolism, 1977. **45**(4): p. 768-774.

21. St John, A.L. and A.P.S. Rathore, *Early Insights into Immune Responses during COVID-19.* 2020.

22. Wu, Z. and J.M. McGoogan, *Characteristics of and important lessons from the coronavirus disease 2019 (COVID-19) outbreak in China: summary of a report of 72 314 cases from the Chinese Center for Disease Control and Prevention.* Jama, 2020. **323**(13): p. 1239-1242.

23. Chu, H., et al., *Middle East respiratory syndrome coronavirus efficiently infects human primary T lymphocytes and activates the extrinsic and intrinsic apoptosis pathways.* The Journal of infectious diseases, 2016. **213**(6): p. 904-914.

24. Law, H.K., et al., *Chemokine up-regulation in sars-coronavirus–infected, monocyte-derived human dendritic cells.* Blood, 2005. **106**(7): p. 2366-2374.

25. Cheung, C.Y., et al., *Cytokine responses in severe acute respiratory syndrome coronavirus-infected macrophages in vitro: possible relevance to pathogenesis*. Journal of virology, 2005. **79**(12): p. 7819-7826.

26. Giménez, V.M.M., et al., *Lungs as target of COVID-19 infection: Protective common molecular mechanisms of vitamin D and melatonin as a new potential synergistic treatment.* Life Sciences, 2020: p. 117808.

27. Ben-Nathan, D., et al., *Protective effects of melatonin in mice infected with encephalitis viruses.* Archives of virology, 1995. **140**(2): p. 223-230.

28. Carrasco, C., et al., *Anti-inflammatory effects of melatonin in a rat model of caerulein-induced acute pancreatitis.* Cell Biochem Funct, 2013. **31**(7): p. 585-90.

29. Pourhanifeh, M.H., et al., *Melatonin: new insights on its therapeutic properties in diabetic complications.* Diabetol Metab Syndr, 2020. **12**: p. 30.

30. Coppola, S., A. Caccioppola, and D. Chiumello, *Internal clock and the surgical ICU patient.* Curr Opin Anaesthesiol, 2020. **33**(2): p. 177-184.

31. Akbulut, H.F., H. Vatansev, and T. Sekmenli, *Determination of Melatonin Deprivation Impact on Sepsis With Acute Phase Reactants.* J Surg Res, 2020. 247: p. 108-114.

32. Can, M.G. and H. Ulugöl, *Effects of Alprazolam and Melatonin Used for Premedication on Oxidative Stress, Glicocalyx Integrity and Neurocognitive Functions.* 2018. **46**(3): p. 233-237.

33. Gupta, P., et al., *Role of melatonin in attenuation of haemodynamic responses to laryngoscopy and intubation.* Indian J Anaesth, 2016. **60**(10): p. 712-718.

34. Nickkholgh, A., et al., *The use of high-dose melatonin in liver resection is safe: first clinical experience.* J Pineal Res, 2011. **50**(4): p. 381-8.

35. Norouzi, A., et al., *Premedication effect of melatonin on propofol induction dose for anesthesia, anxiety, orientation and sedation after abdominal surgery: a double-blinded randomized trial.* Med Gas Res, 2019. **9**(2): p. 62-67.

36. Andersen, L.P., et al., *Pharmacokinetics of high-dose intravenous melatonin in humans.* J Clin Pharmacol, 2016. **56**(3): p. 324-9.

37. Galley, H.F., et al., *Melatonin as a potential therapy for sepsis: a phase I dose escalation study and an ex vivo whole blood model under conditions of sepsis.* J Pineal Res, 2014. **56**(4): p. 427-38.

38. Alamili, M., et al., *Melatonin suppresses markers of inflammation and oxidative damage in a human daytime endotoxemia model.* J Crit Care, 2014. **29**(1): p. 184.e9-184.e13.

39. Weishaupt, J.H., et al., *Reduced oxidative damage in ALS by high-dose enteral melatonin treatment.* J Pineal Res, 2006. **41**(4): p. 313-23.
